# DOES RURAL LIVING INCREASE RISK FOR MORTALITY? FINDINGS FROM TWO LONGITUDINAL STUDIES

**DOI:** 10.1093/geroni/igad104.3798

**Published:** 2023-12-21

**Authors:** Olivia Atherton

**Affiliations:** University of Houston, Houston, Texas, United States

## Abstract

Most prior work on rural-urban health disparities has been conducted with population-level data, which is limited in its capacity for causal inferences about individuals and lifespan health. The present study leverages individual-level longitudinal data, spanning up to 29 years, to understand how rurality-urbanicity predicts individuals’ risk for all-cause mortality; whether these effects hold above and beyond socioeconomic status; and whether the association between rurality-urbanicity and mortality risk varies by sex, socioeconomic status, race, and partner status. The present pre-registered study uses data from two large longitudinal studies of U.S. Americans (HRS and MIDUS; total N = ~55,000), who reported on their socio-demographic characteristics, had their addresses linked to geographical indicators (i.e., rural-urban continuum codes), and have data from the National Death Index regarding vital status and survival time. Using a series of cox proportional hazards regression models, findings showed that suburban and rural residents were at a 12% and 18% greater risk for earlier all-cause mortality compared to urban residents in HRS, but the effects of rurality-urbanicity on mortality risk were non-significant in MIDUS. The longitudinal effects of rurality-urbanicity on mortality risk were largely independent of the effects of socioeconomic status. Finally, the three statistically significant interaction effects suggest that the strength and direction of the association between rurality-urbanicity and mortality risk differed by race (HRS, MIDUS) and partner status (HRS). The discussion elaborates on the theoretical implications for our understanding of social determinants of health, as well as practical implications for health equity in rural areas.

